# Paltusotine versus octreotide: different effects on radioligand uptake in neuroendocrine tumours

**DOI:** 10.1530/EO-25-0041

**Published:** 2025-10-18

**Authors:** Katharina Wang, Christoph J Auernhammer, Simon Lindner, Mathias Zacherl, Rudolf A Werner, Julian Maurer, Lea Peischer, Julia Hamati, Maximilian P Hungbauer, Thomas Knösel, Astrid Reul, Elena Kuzmenko, Diana Vetter, José Oberholzer, Umberto Maccio, Felix Beuschlein, Martin Reincke, Constanze Hantel, Karel Pacak, Ashley B Grossman, Kathrin Zitzmann, Svenja Nölting

**Affiliations:** ^1^ Department of Internal Medicine IV, LMU University Hospital, LMU Munich, Munich, Germany; ^2^ Interdisciplinary Center of Neuroendocrine Tumors of The GastroEnteroPancreatic System (GEPNET-KUM, ENETS Centre of Excellence), LMU University Hospital, Munich, Germany; ^3^ Department of Nuclear Medicine, LMU University Hospital, LMU Munich, Munich, Germany; ^4^ Department of General, Visceral and Transplantation Surgery, LMU University Hospital, LMU Munich, Munich, Germany; ^5^ Institute of Pathology, Faculty of Medicine, LMU Munich, Munich, Germany; ^6^ Department of Endocrinology, Diabetology and Clinical Nutrition, University Hospital Zurich, Zurich, Switzerland; ^7^ Department of Visceral and Transplantation Surgery, University Hospital Zurich, Zurich, Switzerland; ^8^ Department of Pathology and Molecular Pathology, University Hospital Zurich, Zurich, Switzerland; ^9^ The LOOP Zurich – Medical Research Center, Zurich, Switzerland; ^10^ AKESO, Prague, Czech Republic; ^11^ Palacky University, Olomouc, Czech Republic; ^12^ Green Templeton College, University of Oxford, Oxford, UK; ^13^ NET Unit, ENETS Centre of Excellence, Royal Free Hospital, London, UK; ^14^ ENETS Centre of Excellence Zurich, University Hospital Zurich, Zurich, Switzerland

**Keywords:** paltusotine, octreotide, somatostatin analogue, neuroendocrine tumour, peptide receptor radionuclide therapy, PRRT

## Abstract

**Objective:**

Somatostatin receptor analogues are well-established in the treatment of metastatic gastro-enteropancreatic neuroendocrine tumours (GEP-NETs), especially for symptom control in patients with the carcinoid syndrome, and to control tumour growth. However, they need to be discontinued before peptide receptor radionuclide therapy (PRRT) as they may saturate the somatostatin receptor 2 (SSTR2) and prevent binding of the radioactive ligand.

**Design:**

We evaluated the effects of the novel somatostatin analogue paltusotine on ^18^F-SiTATE radioligand uptake and on GEP-NET cell viability in comparison to octreotide.

**Methods:**

Paltusotine and octreotide were evaluated in varying concentrations in an ^18^F-SiTATE uptake assay using stable hSSTR2 over-expressing BON-1 cells, and in a cell viability assay utilising different NET cell lines and human patient-derived GEP-NET primary cultures (*n* = 13).

**Results:**

Low, clinically-relevant concentrations of paltusotine (7.3–25.4 nM) demonstrated no influence on cellular radioligand uptake compared to the control. In contrast, octreotide reduced radioligand uptake at low, clinically-relevant concentrations (7.3–25.4 nM) and led to a further significant reduction of radioligand uptake at higher concentrations (73–508 nM). Both paltusotine and octreotide showed overall little or no significant anti-tumour effects *in vitro* in NET cell lines. However, in contrast to octreotide, paltusotine led to a slight decrease in cell viability of patient-derived GEP-NET primary cultures.

**Conclusions:**

Treatment with paltusotine did not significantly reduce radioligand binding of ^18^F-SiTATE *in vitro*, indicating no influence on SSTR2 targeting. This might enable a continuation of somatostatin receptor analogue therapy with paltusotine during PRRT, potentially improving symptom control in GEP-NET patients with the carcinoid syndrome.

## Introduction

Gastro-enteropancreatic neuroendocrine tumours (GEP-NETs) include tumours arising from the pancreas (panNET) and the small intestine (siNET). The latter can present with the carcinoid syndrome, a hormone hypersecretion syndrome characterised by excess serotonin or its metabolites, and accompanied by typical clinical signs and symptoms such as diarrhoea, flushing, bronchospasm and carcinoid heart disease (1).

Somatostatin receptor analogues (SSAs) octreotide and lanreotide are established therapies in the treatment of metastatic GEP-NETs (2, 3). SSAs are used for symptom control in GEP-NET patients with the carcinoid syndrome or for growth control in GEP-NETs with low proliferation rates (Ki-67 of 10% or lower), particularly as first-line therapy (4, 5, 6). However, long-acting SSAs (octreotide LAR, lanreotide autogel) are required to be discontinued 4–6 weeks before initiation of somatostatin receptor- (SSTR-)/peptide receptor-targeted radionuclide therapy (PRRT) as they might negatively influence SSTR targeting by saturation of the SSTR2 (7, 8, 9). For symptom control, short-acting octreotide can be used until 24–48 h before PRRT (7, 9). Moreover, both octreotide and lanreotide are injected subcutaneously or intramuscularly, complicating the application and requiring regular visits to healthcare offices.

A newly developed, orally-bioavailable non-peptide SSTR2 agonist, paltusotine, might simplify SSA therapy and is taken orally once daily (10). Paltusotine recently received FDA approval for acromegaly treatment based on data from two phase 3 trials (PATHFNDR-1 and PATHFNDR-2/NCT05192382) (11, 12). Currently, it is also being investigated in NET patients with the carcinoid syndrome in a randomised phase 2 study (NCT05361668), with preliminary results showing good tolerability and improved symptom control (13). Based on these results, a phase 3 clinical trial evaluating paltusotine in patients with the carcinoid syndrome is currently being planned (CAREFNDR) (14). However, the effects of paltusotine on SSTR2 targeting before PRRT remain largely unexplored.

Therefore, we have investigated the effects of paltusotine in comparison to octreotide on ^18^F-SiTATE radioligand uptake using a stable hSSTR2 over-expressing BON-1 cell clone (BON-1 SSTR2), as well as the efficacy of paltusotine in GEP-NET cell lines and human patient-derived GEP-NET primary cultures (*n* = 13).

## Materials and methods

### NET cell lines and GEP-NET primary cultures

The BON-1 (human panNET; Prof. R Göke, University of Marburg, Germany), NCI-H727 (human bronchopulmonary NET; ATCC, USA) and QGP-1 cell lines (human panNET; JCRB Cell Bank, Japan) were cultured as previously described (15). Moreover, a stable hSSTR2 over-expressing BON-1 cell clone (BON-1 SSTR2) was generated as previously described (16, 17).

Fresh patient-derived GEP-NET primary cultures were established from surgically-derived patient tumours as previously described (18). In total, 15 primary cultures were generated from 14 patients. Eleven of these were metastatic GEP-NETs, including six siNETs (*n* = 2 with the carcinoid syndrome) and eight panNETs. The use of primary tumour tissue as part of NeoExNET was approved by the Ethics Committee of LMU Munich (project number 152-10) and by the Cantonal Ethics Committee Zurich (reference number BASEC 2017-00950).

### Dose finding of SSAs

Paltusotine and octreotide (both MedChemExpress, USA) were tested in concentrations of 4–500 nM. Dose-finding of clinically-relevant concentrations was performed by identifying the maximum plasma concentrations found in humans after treatment with the respective substances. Single doses of paltusotine led to plasma concentrations of around 22–406 nM in healthy volunteers (19). Subcutaneous octreotide injections led to plasma concentrations of up to 4 nM (20). In GEP-NET primary cultures, supraphysiological concentrations of 40 μM octreotide were additionally tested since lower concentrations were ineffective.

### Cell viability assay

GEP-NET cell lines and primary cultures were treated for 72 h with either paltusotine or octreotide, and dimethyl sulfoxide (DMSO) was used as control. Cell Titer Blue® cell viability assay (Promega, USA) was then performed as previously described (15, 21). Each experiment included 3–4 samples per drug concentration and patient. Cell line experiments were performed at least three times.

### ^18^F-SiTATE uptake assay

For the ^18^F-SiTATE uptake assay, BON-1 SSTR2 cells were seeded into 6-well plates (Sarstedt, German) with DMEM/F12 culture medium (Thermo Fisher Scientific, USA), supplemented with 10% FCS, 1% penicillin/streptomycin (all from Thermo Fisher Scientific) and 0.4% amphotericin B (PAN-Biotech, Germany). On the day of the experiments, the culture medium was exchanged with 600 μL fresh medium. 8kBq of ^18^F-SiTATE per 200 μL culture medium was calculated based on the freshly prepared daily stock provided by the Department of Nuclear Medicine. 200 μL containing 8kBq ^18^F-SiTATE with or without blocking substances (octreotide, paltusotine, DOTATATE acetate (ABX, Germany)) was then added into the corresponding wells and incubated for 30, 120 and 240 min. In order to provide a direct comparison of therapy versus the unblocked control, 10-fold, 100-fold and 200-fold the amount of substance of paltusotine or octreotide compared to ^18^F-SiTATE were used (100×: 7.3–25.4 nM, 1,000×: 73–254 nM, 2,000×: 146–508 nM). A 1,000-fold excess of unlabelled DOTATATE acetate was used as a blocked control for determination of non-specific internalisation. Untreated cells were used as an unblocked control to determine total cellular uptake. After discarding the supernatant, the cells were washed with 800 μL PBS (Merck, Germany) and treated twice with 800 μL 2 M sodium hydroxide (NaOH; Carl Roth, Germany) for 5–10 min to detach the cells from the plates. The supernatant was then collected (in total, 1.6 mL) and radioactivity was measured as previously described (17) using the Hidex Automatic Gamma Counter (Hidex Oy, Finland). Results were expressed as the percentage of measured to total added radioactivity and corrected to the rate of ^18^F-SiTATE decay (by normalising to the time at which ^18^F-SiTATE was added to the wells). After performing the experiments, the radioactive substances were discarded following the standard protocol of the Department of Nuclear Medicine. A total of four independent experiments with two technical replicates (wells) per drug concentration were performed.

### Statistical analysis

Testing for normal distribution was performed using the Shapiro–Wilk test. In the case of parametric data, one-way ANOVA was performed followed by *post hoc* Dunnett’s test for comparison of treatment groups with the control group. For non-parametric data, the Kruskal–Wallis test was performed followed by a *post hoc* test with Bonferroni correction. A significance level of *P* < 0.05 was used. Statistical analysis was performed using IBM SPSS Statistics, Version 29.0.2.0 (IBM Corp., USA, released 2023). Due to the exploratory nature of the study, our data should be interpreted purely descriptively.

## Results

### Effects of paltusotine vs octreotide on ^18^F-SiTATE uptake in BON-1 SSTR2 cells

**Figure 1:**
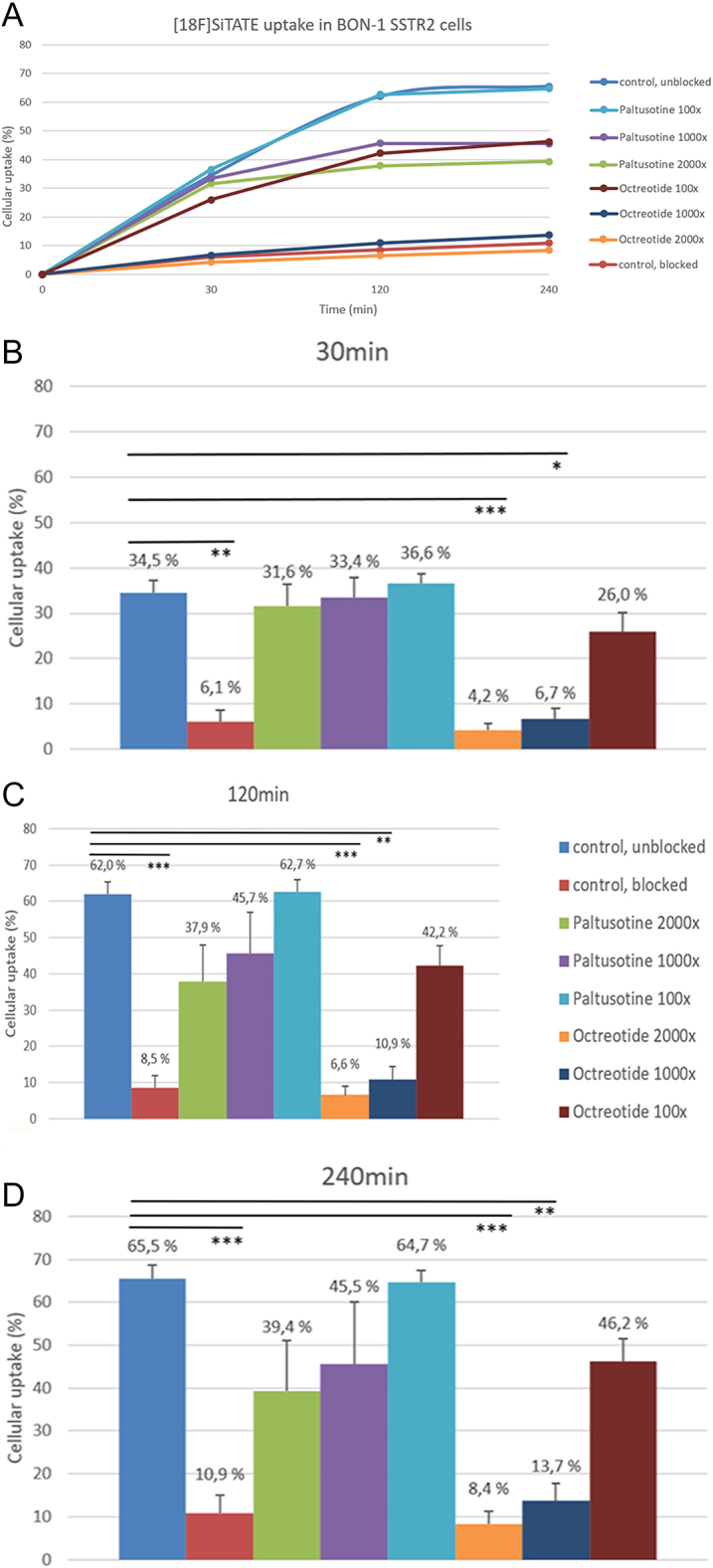
(A) ^18^F-SiTATE uptake assay using BON-1 SSTR2 cells after treatment with either paltusotine or octreotide. Cellular uptake was measured after (B) 30 min, (C) 120 min and (D) 240 min. DOTATATE acetate was used as the blocked control. The data are depicted as mean ± SD of four duplicate experiments. Molar concentrations: 100× (7.3–25.4 nM), 1,000× (73–254 nM), 2,000× (146–508 nM). Significantly lower radioligand uptake compared to the unblocked control *P < 0.05, **P < 0.01, ***P < 0.001.

In order to evaluate the cellular uptake of ^18^F-SiTATE, we utilised BON-1 SSTR2 cells, which highly express SSTR2. Total cellular uptake of ^18^F-SiTATE in BON-1 SSTR2 cells (unblocked control) was 34.5% ± 2.8% after 30 min and reached a plateau after 120 and 240 min at 62% ± 3.3 and 65.5% ± 3.1% (Fig. 1). Incubation with paltusotine at clinically-relevant concentrations led to little to no reduction of ^18^F-SiTATE radioligand uptake: paltusotine at low, clinically-relevant concentrations (7.3–25.4 nM (100×)) showed no influence on radioligand binding compared to the unblocked control, with 36.6% ± 2.1%, 62.7% ± 3.2 and 64.7% ± 2.8% uptake after 30, 120 and 240 min, respectively. Even at higher clinically-relevant concentrations of paltusotine, no significant effects on cellular uptake were observed compared to the unblocked control. With paltusotine (73–254 nM (1,000×) and 146–508 nM (2,000×)), cellular uptake was still 33.4% ± 4.4%, 45.7% ± 11.2%, 45.5% ± 14.5 and 31.6% ± 4.8%, 37.9% ± 10.1%, 39.4% ± 11.7%, respectively, after 30, 120 and 240 min.

In contrast, treatment with octreotide (73–254 nM (1,000×) and 146–508 nM (2,000×)) significantly reduced cellular uptake compared to the unblocked control (Supplementary Table 1 (see section on Supplementary materials given at the end of the article)), leading to 6.7% ± 2.3%, 10.9% ± 3.6%, 13.7% ± 4.2% uptake at 1,000× and 4.2% ± 1.6%, 6.6% ± 2.3%, 8.4% ± 2.9% uptake at 2,000×, respectively, after 30, 120 and 240 min. Low concentrations of octreotide (7.3–25.4 nM (100×)) led to 26% ± 4.2%, 42.2% ± 5.5 and 46.2% ± 5.3% uptake after 30, 120 and 240 min.

### Effects of paltusotine vs octreotide on cell viability in NET cell lines and primary cultures

**Figure 2:**
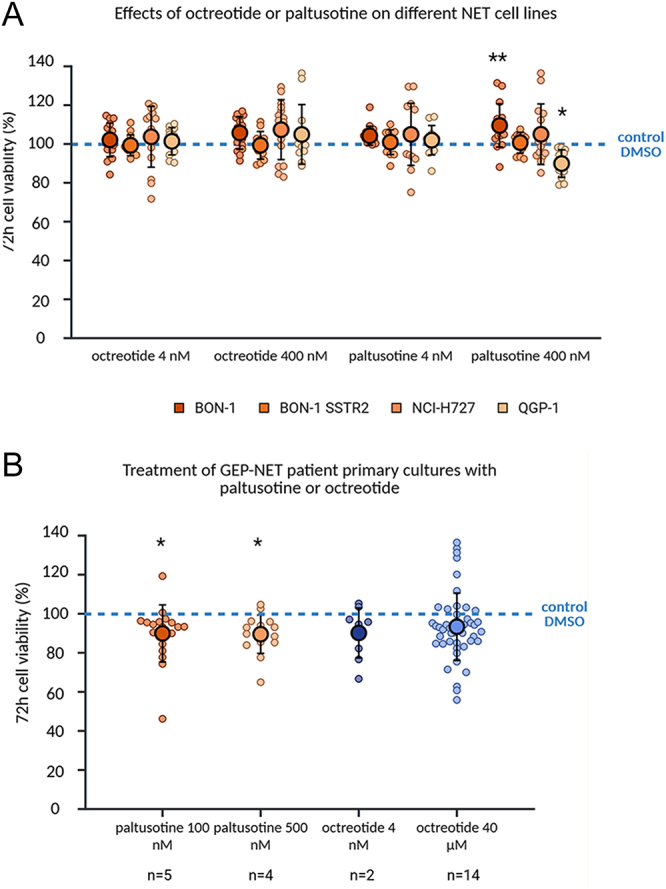
Treatment of different (A) NET cell lines and (B) patient-derived GEP-NET primary cultures (*n* = 14) with paltusotine and octreotide. Each NET cell line is depicted in a different colour. The data are depicted as mean ± SD including the individual data points. Significant increase/decrease in cell viability compared to control DMSO *P < 0.05, **P < 0.01, ***P < 0.001.

Both octreotide (4 nM, 400 nM) and paltusotine (4 nM) showed overall no anti-tumour effects on different NET cell lines (BON-1, BON-1 SSTR2, NCI-H727, QGP-1) *in vitro* (Fig. 2A). Paltusotine (400 nM) led to a slight, significant increase in viability in BON-1 cells and to a slight, significant decrease in cell viability in QGP-1 cells. These effects were not found in the other cell lines or drug concentrations.

**Figure 3:**
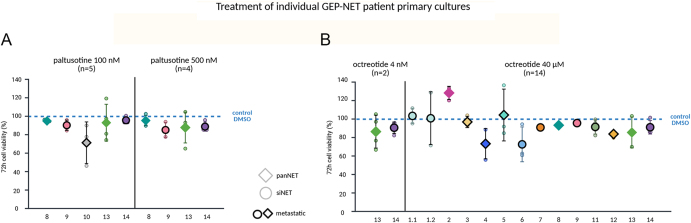
Individual patient-derived GEP-NET primary cultures after incubation with (A) paltusotine 100 and 500 nM or (B) octreotide 4 nM and 40 μM. panNETs are diamond-shaped and siNETs are circular-shaped. Each primary culture is shown in a different colour; metastatic tumours are depicted with a black frame. The data are depicted as mean ± SD including the individual data points.

In patient-derived GEP-NET primary cultures, neither clinically-relevant nor higher concentrations of octreotide (4 nM, 40 μM, *n* = 14) displayed any overall effects on cell viability, while paltusotine (100 nM, 500 nM, *n* = 5) showed overall slight anti-tumour effects (Fig. 2B). Individual patient primary culture responses to treatment with paltusotine or octreotide are shown in Fig. 3. The detailed patient characteristics of these GEP-NET primary cultures are listed in Table 1.

**Table 1:** Patient characteristics of GEP-NET primary cultures.

Patient ID*	Sex	Age (years)	Grading (WHO)	Tumour type	Metastatic	Tumour characteristics, histology	Ki-67	Patient characteristics	Functionally active
1.1	f	56	NET G1	siNET	Yes	Primary tumour (ileum), max 1.6 cm, ulceration of mucosa, muscularis propria invasion	<2%	Metastatic (liver, ovaries, peritoneal, lymph node), no clinical symptoms	Carcinoid syndrome
1.2						Metastasis (peritoneal), max 1.7 cm		Same patient as 1.1	
2	f	38	NET G2	panNET	No	Primary tumour, max 3.4 cm	3–4%	No clinical symptoms	No
3	f	64	NET G2	panNET	Yes	Metastasis (lymph node), max. 2.9 cm, capsular infiltration, ISLET1 positivity	15%	Metastatic (lymph nodes), chronic diarrhoea	No
4	f	55	NET G1	panNET	Yes	Primary tumour, max 9.2 cm, angioinvasion, infiltration of spleen and stomach, lymphatic infiltration	2%	Metastatic (liver, lymph node), upper abdominal pain, no other clinical symptoms	No
5	m	72	NET G2	panNET	Yes	Metastasis (liver), max 2.3 cm	5–10%	Metastatic (liver, lymph node)	No
6	f	63	NET G1	siNET	Yes	Metastasis (liver), max 7.5 cm, lymphatic infiltration	<1%	Metastatic (liver, lymph node), chronic diarrhoea, flush, hot flashes, weight loss, lipoedema/lymphedema	Carcinoid syndrome
7	m	63	NET G1	siNET	Yes	Metastasis (liver), max. 2.6 cm, lymphatic infiltration	1%	Metastatic (liver, lymph nodes), concurrent well differentiated pheochromocytoma 1.6 cm	No
8	f	36	NET G1	panNET	No	Primary tumour, 3.4 cm	1%	Nausea, abdominal pain	No
9	m	70	NET G2	siNET	Yes	Primary tumour, max 1.6 cm	5%	Metastatic (lymph node)	No
10	m	50	NET G2	panNET	Yes	Primary tumour, 3.8 cm	15%	Metastatic (liver, lung)	No
11	m	65	NET G1	siNET	Yes	Metastasis (lymph node), 10 mm, SSTR2 positive	2%	Metastatic (lymph node)	No
12	m	61	NET G2	panNET	Yes	Primary tumour, 6.5 cm, SSTR2 positive	5%	Metastatic (lymph node)	No
13	m	72	NET G2	panNET	No	Primary tumour, 9.6 cm	15%	No clinical symptoms	No
14	f	52	NET G2	siNET	Yes	Metastasis (liver), max. 2.6 cm	15%	Metastatic (liver, lymph node), recurrent abdominal colics, nausea, diarrhoea	Gastrinoma

* Characteristics have in part been published previously (18, 41).

Abbreviations: GEP-NET, gastro-enteropancreatic neuroendocrine tumour; f, female; m, male; siNET, small intestinal NET; panNET, pancreatic NET.

## Discussion

SSAs are established and effective therapies for metastatic GEP-NETs and are essential for symptom control in patients with the carcinoid syndrome. However, according to current guidelines as well as the FDA/EMA labelling instructions regarding Lutathera®, SSA therapy needs to be discontinued before PRRT (7, 8, 9). These recommendations stem from the fact that both SSAs and PRRT target the SSTR2. Octreotide and lanreotide primarily bind to SSTR2 and show reduced or no binding affinity to SSTR1/3/4/5 (22). Moreover, long-acting octreotide and prolonged-release lanreotide maintain plateau-level concentrations for about 30 days (octreotide), or reach initial peak concentrations on day 1 followed by a consistent decrease throughout the treatment period with a half-life of 25.5 days (lanreotide) (23). Therefore, long-acting SSAs need to be discontinued at least 4 weeks before the application of PRRT (7, 8, 9). Some authors suggest no interference of SSAs with PRRT (24). However, currently, no data exist, including studies investigating SSA/PRRT combination therapies, which clearly show that long-acting SSA injection intervals <4–6 weeks before PRRT would not interfere with PRRT (24, 25, 26, 27, 28, 29). In contrast to PRRT, SSAs do not need to be paused before SSTR PET/CT imaging, as the tumour-to-background ratio is essential in this setting (30, 31, 32).

The temporary discontinuation of SSAs before PRRT may be especially problematic for GEP-NET patients suffering from strong carcinoid syndrome-related symptoms. Moreover, there is clinical interest in SSA/PRRT combination therapy (26, 27, 28, 29). Thus, an ongoing prospective clinical trial is addressing the unanswered question as to whether SSAs really need to be paused for several weeks before PRRT (NCT06855095).

Therefore, the effects of a novel oral non-peptide SSTR2-specific agonist, paltusotine, are now particularly relevant. The SSTR2 binding affinity of paltusotine is similar to that of somatostatin, octreotide and lanreotide, with IC50 of 0.25 versus 0.15, 0.38 and 0.54 nM, respectively (33). In addition, paltusotine showed similar efficacy as octreotide in inhibition of forskolin-stimulated cAMP accumulation, with an EC50 of 2.08 ± 1.39 nM vs 0.21 ± 1.35 nM (34).

While G-protein (GP)- and arrestin (ARRB)-mediated effects are induced by full SSTR2 agonists such as octreotide (35), biased agonists with GP-mediated effects but diminished ARRB signalling have been reported (35). Arrestins play an important role in SSTR internalisation and recycling/degradation (35). Zhao *et al.* compared the molecular binding structure of octreotide and paltusotine to SSTR2 and their molecular signalling, examined β-arrestin recruitment and SSTR2 internalisation/loss of SSTR2 expression on the cell surface of HEK293 cells (34). Octreotide potently induced β-arrestin recruitment and caused SSTR2 internalisation/desensitisation (34). In contrast, paltusotine caused much lower β-arrestin recruitment and less SSTR2 internalisation/desensitisation (34).

Based on these preclinical mechanistic findings (34), we compared ^18^F-SiTATE radioligand uptake between NET cells treated with paltusotine or octreotide. Importantly, clinically-relevant concentrations of paltusotine (7.3–25.4 nM) did not influence radioligand uptake of ^18^F-SiTATE in hSSTR2 over-expressing BON-1 cells compared to the unblocked control; in contrast, octreotide significantly reduced radioligand uptake compared to the unblocked control. Thus, from a translational perspective, our findings might have clinical implications for treatment of patients with carcinoid syndrome during PRRT, potentially enabling a continuation of symptom control using paltusotine, if confirmed by independent approaches.

Paltusotine is currently being investigated in a randomised phase 2 trial in NET patients with the carcinoid syndrome (NCT05361668), with promising preliminary results (13), and a phase 3 trial is currently being planned (14). Moreover, the good oral bioavailability of paltusotine (10) in contrast to octreotide (36) may increase the quality of life of NET patients by reducing the frequency of healthcare office visits and eliminating injection-associated pain (10, 37). Recently, paltusotine received FDA approval for the treatment of patients with acromegaly (11, 12).

It should be further investigated in future studies whether paltusotine also demonstrates antiproliferative/apoptotic efficacy in NET cells. While ARRB2 signalling has been reviewed to be essential for antiproliferative effects (35), paltusotine seems to demonstrate a biased agonism with highly efficacious GP-mediated effects but diminished ARRB signalling in comparison to octreotide (34). Nevertheless, in pituitary tumour cells, paltusotine demonstrated an increased apoptotic rate compared to octreotide (34).

Since displacement of the radioligand occurs at the SSTR2 binding pocket and thus upstream of ARRB- or GP-signalling, a link between paltusotine signalling and its low ^18^F-SiTATE displacement will still need to be shown and further explored. We are aware of the unexpectedly large difference in radioligand uptake between paltusotine and octreotide in hSSTR2 over-expressing BON-1 cells, given that they share similar affinity and binding sites. Therefore, our data should be considered strictly preliminary before confirmation independently by alternative methods.

In NET cell lines, we found overall no anti-tumour effects of paltusotine or octreotide at clinically-relevant or supraphysiological concentrations, with only 400 nM paltusotine showing slight anti-tumour efficacy in QGP-1 cells. However, in patient-derived GEP-NET primary cultures, paltusotine displayed a slight overall anti-tumour efficacy, potentially indicating slightly improved efficacy of paltusotine compared to octreotide. Consistently, previous *in vitro* studies showed no significant growth inhibition of octreotide in NET cell lines or phaeochromocytoma/paraganglioma primary cultures (38, 39, 40). In contrast, the efficacy of SSAs *in vivo* in patients with GEP-NETs is well established (2, 3). Therefore, the efficacy of SSAs may be due to a different mode of action than by direct targeting of tumour cells (38) or due to stabilisation of disease but not tumour cell death. However, these effects, including potential effects on the tumour microenvironment or on disease stabilisation (an important mode of action of SSAs), cannot be investigated in our *in vitro* study. The absence of confirmation by an independent method is another limitation. Therefore, these data should be regarded as preliminary.

## Conclusions

In conclusion, we provide novel data on the effects of paltusotine on ^18^F-SiTATE radioligand uptake in GEP-NET cells. In contrast to octreotide, paltusotine displayed no influence on SSTR2 receptor binding at clinically-relevant concentrations. These preliminary data might be of high interest to GEP-NET patients with the carcinoid syndrome and, if confirmed *in vivo*, could allow for a continuation of SSA therapy during PRRT and improve symptom and tumour growth control in these patients.

## Supplementary materials



## Declaration of interest

RAW: speaker honoraria from Novartis/AAA and PentixaPharm, advisory board work for Novartis/AAA and Bayer. MR has received honoraria from Crinetics as a member of the Scientific Advisory Board.

## Funding

This work was supported by the German Research Foundation (Deutsche Forschungsgemeinschaft): CRC/Transregio 205/2, Project number: 314061271–TRR 205 ‘The Adrenal: Central Relay in Health and Disease’.

## Author contribution statement

CJA and SN conceptualised the study. CJA, SL, MZ, RAW and SN were responsible for methodology. KW, CJA and SN provided validation. KW helped in formal analysis. JM, LP, JH, AR and EK contributed to investigation. CJA, SL, MZ, RAW, MPH, TK, DV, JO, UM and SN provided resources. KW, CJA and SN wrote the original draft. JO, FB, MR, CH, KP, ABG and KZ were responsible for writing review and editing. KW and JM provided visualisation. CJA and SN supervised the study. KW, CJA and SN administered the project. SN was responsible for funding acquisition. All authors have read and agreed to the published version of the manuscript.
