# Supplementary material for: Paltusotine versus octreotide: different effects on radioligand uptake in neuroendocrine tumours
Source: Endocr Oncol. 2025 Oct 18;5(1):e250041. doi: 10.1530/EO-25-0041 (PMC12538274; doi:10.1530/EO-25-0041)
Supplement: Supplementary file 1 [file supplementary_materials.pdf]

Supplementary table 1: Multiple comparison of [^{18}F]SiTATE uptake after different treatments and measured after 30min

	control, unblocked	control, blocked	paltusotine 2000x	paltusotine 1000x	paltusotine 100x	octreotide 2000x	octreotide 1000x	octreotide 100x
control, unblocked	-	**	ns	ns	ns	***	*	ns
control, blocked	**	-	ns	**	***	ns	ns	ns
paltusotine 2000x	ns	ns	-	ns	ns	**	ns	ns
paltusotine 1000x	ns	**	ns	-	ns	**	*	ns
paltusotine 100x	ns	***	ns	ns	-	***	**	ns
octreotide 2000x	***	ns	**	**	***	-	ns	ns
octreotide 1000x	*	ns	ns	*	**	ns	-	ns
octreotide 100x	ns	ns	ns	ns	ns	ns	ns	-

*p<0.05, **p<0.01, ***p<0.001, ns non-significant

Supplementary table 2: Multiple comparison of [^{18}F]SiTATE uptake after different treatments and measured after 120min

	control, unblocked	control, blocked	paltusotine 2000x	paltusotine 1000x	paltusotine 100x	octreotide 2000x	octreotide 1000x	octreotide 100x
control, unblocked	-	***	ns	ns	ns	***	**	ns
control, blocked	***	-	ns	ns	***	ns	ns	ns
paltusotine 2000x	ns	ns	-	ns	ns	ns	ns	ns
paltusotine 1000x	ns	ns	ns	-		*	ns	ns
paltusotine 100x	ns	***	ns	ns	-	***	**	ns
octreotide 2000x	***	ns	ns	*	***	-	ns	ns
octreotide 1000x	**	ns	ns	ns	**	ns	-	ns
octreotide 100x	ns	ns	ns	ns	ns	ns	ns	-

*p<0.05, **p<0.01, ***p<0.001, ns non-significant

Supplementary table 3: Multiple comparison of [^{18}F]SiTATE uptake after different treatments and measured after 240min

	control, unblocked	control, blocked	paltusotine 2000x	paltusotine 1000x	paltusotine 100x	octreotide 2000x	octreotide 1000x	octreotide 100x
control, unblocked	-	***	ns	ns	ns	***	**	ns
control, blocked	***	-	ns	ns	***	ns	ns	ns
paltusotine 2000x	ns	ns	-	ns	ns	ns	ns	ns
paltusotine 1000x	ns	ns	ns	-	ns	*	ns	ns
paltusotine 100x	ns	***	ns	ns	-	***	**	ns
octreotide 2000x	***	ns	ns	*	***	-	ns	*
octreotide 1000x	**	ns	ns	ns	**	ns	-	ns
octreotide 100x	ns	ns	ns	ns	ns	*	ns	-

*p<0.05, **p<0.01, ***p<0.001, ns non-significant
